# Collision metastasis in a pelvic lymph node from urothelial and prostatic carcinoma: a rare case report and literature review

**DOI:** 10.3389/fonc.2025.1658290

**Published:** 2025-12-04

**Authors:** Xue-Dong Shi, Qiong-Nan Shen, Li Wei, Li-Juan Chen, Hui-Fen Wang, Xu-Dong Zheng, Qi Ma

**Affiliations:** 1 Department of Urology, The Affiliated Yangming Hospital of Ningbo University, Ningbo, Zhejiang, China; 2 Health Science Center, Ningbo University, Ningbo, Zhejiang, China; 3 Department of Pathology, The Affiliated Yangming Hospital of Ningbo University, Ningbo, Zhejiang, China; 4 Comprehensive Genitourinary Cancer Center, The First Affiliated Hospital of Ningbo University, Ningbo, Zhejiang, China; 5 Yi-Huan Genitourinary Cancer Group, The First Affiliated Hospital of Ningbo University, Ningbo, Zhejiang, China

**Keywords:** collision metastasis, urothelial carcinoma, prostatic adenocarcinoma, liver metastasis, bone metastasis

## Abstract

Metastasis of both urothelial carcinoma and prostatic carcinoma to the same lymph node is extremely rare. We report a case of collision metastasis involving bladder urothelial carcinoma and prostatic adenocarcinoma in a 76-year-old male. The patient underwent laparoscopic radical cystoprostatectomy and bilateral pelvic lymph node dissection. Final pathological examination revealed high-grade urothelial carcinoma (pT2bN1M0) and prostatic adenocarcinoma (pT2N1M0, Gleason score 4 + 4 = 8). Notably, coexisting metastatic foci of urothelial carcinoma and prostatic adenocarcinoma were identified within a single pelvic lymph node. During the 18-month postoperative follow-up, the patient developed multifocal hepatic and bone metastases. Collision metastasis is a rare clinical event with complex prognostic implications. This study aims to enhance understanding of this unique pathological entity and provide clinical references for the management of such cases through case analysis and literature review.

## 1 Introduction

Collision metastasis refers to the coexistence of metastatic deposits from two independent primary tumors with distinct histological features at the same anatomical site (1). The simultaneous presence of prostatic adenocarcinoma in radical cystectomy specimens for bladder cancer is not uncommon. However, reports in the literature of simultaneous metastasis of urothelial carcinoma and prostatic carcinoma in a single lymph node are extremely rare. To our knowledge, only seven cases have been recorded. Among these cases, six were identified during radical cystoprostatectomy with pelvic lymphadenectomy, while one was detected during right nephroureterectomy with periureteral lymph node dissection. This study reports a case of lymph node collision metastasis involving both urothelial carcinoma and prostatic adenocarcinoma. As far as we know, this is the first case from China.

## 2 Case report

### 2.1 Lymph node metastases of both urothelial carcinoma and prostatic adenocarcinoma

The patient was a 73-year-old Chinese male (76 years old at the time of lymph node collision metastasis diagnosis) with a history of laparoscopic cholecystectomy and one episode of gadolinium contrast-induced syncope, meeting criteria for grade III acute hypersensitivity reaction. The patient had no family history of urological tumors. Additionally, the patient experienced three episodes of acute epididymitis within one year before urothelial carcinoma diagnosis. In March 2020, the patient presented with gross hematuria, and ultrasonography revealed a bladder mass. Following hospitalization, cystoscopy and contrast-enhanced CT Urography (CTU) identified a 9 × 9 mm nodule on the left posterior bladder wall. The patient underwent transurethral bladder tumor resection and subsequent pathological analysis revealed high-grade muscular invasive urothelial carcinoma (pT2b) characterized by localized glandular differentiation. A radical cystectomy (RC) was recommended to this patient, however, after being fully informed, the patient expressed concerns about the extensive surgical trauma and potential decline in postoperative quality of life, and refused RC at that time and asked for regular postoperative intravesical chemotherapy with close surveillance. During follow-up in September 2023, cystoscopy revealed a mucosal protrusion on the left bladder wall, prompting repeat transurethral resection. Pathology confirmed the recurrence of high-grade muscle invasive papillary urothelial carcinoma (pT2) again. However, the patient still declined RC after fully informed.

**Figure 1:**
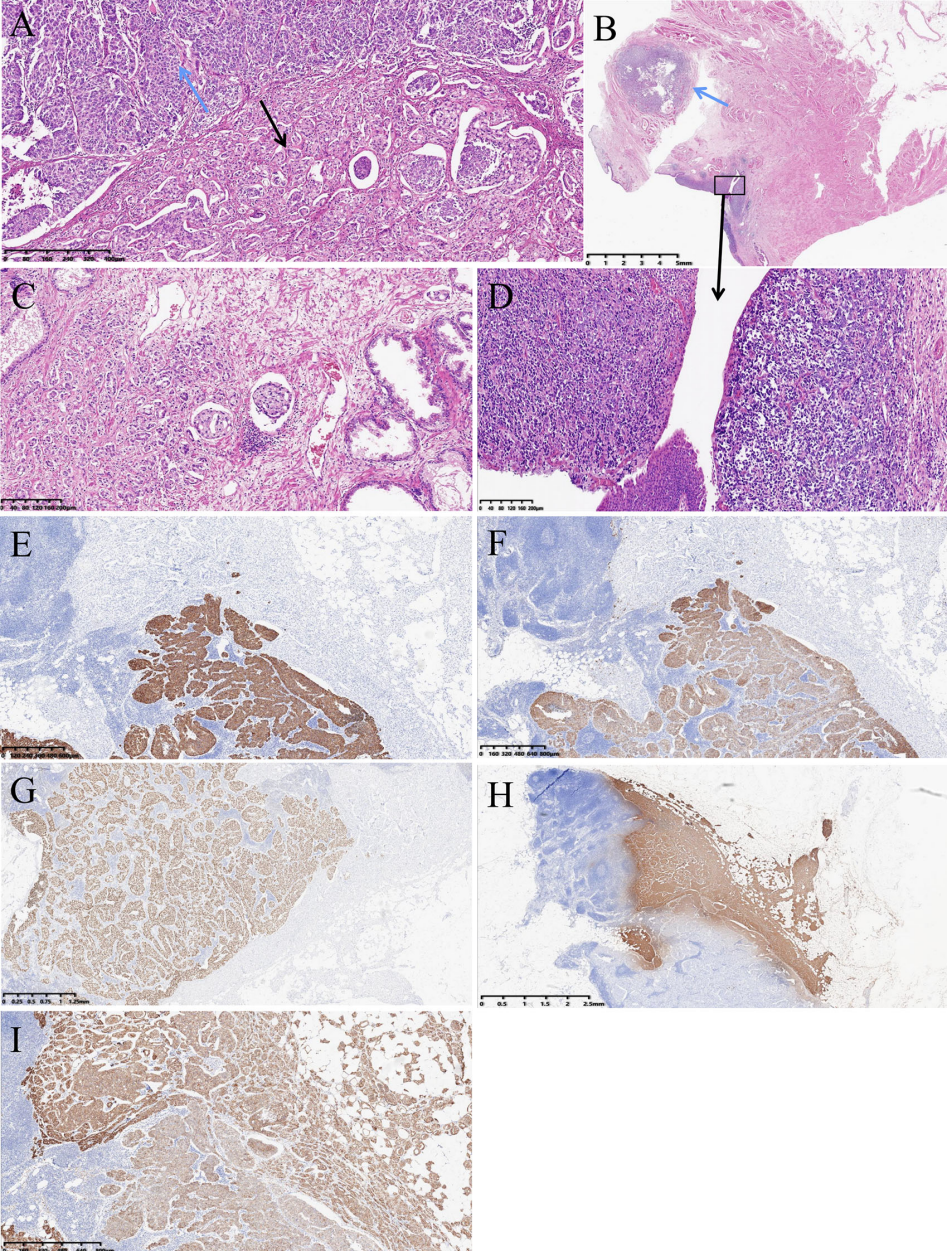
**(A)** Lymph node containing two types of metastases, prostatic adenocarcinoma (black arrow) and urothelial carcinoma (blue arrow),hematoxylin and eosin(HE) stain; **(B)** High-grade urothelial carcinoma with focal glandular differentiation (blue arrow), original tumor, HE stain; **(D)** Urothelial carcinoma under high magnification, HE stain; **(C)** Prostatic adenocarcinoma, original tumor, HE stain; **(E)** Metastatic urothelial carcinoma in the lymph node (cytokeratin 7 stain); **(F)** Metastatic urothelial carcinoma in the lymph node (cytokeratin 20 stain); **(G)** Metastatic urothelial carcinoma in the lymph node (GATA-binding protein 3 stain); **(H)** Metastatic prostatic adenocarcinoma in the lymph node (prostate-specific antigen stain); **(I)** Metastatic urothelial carcinoma and prostatic adenocarcinoma in the lymph node (Alpha-methylacyl-CoA racemase stain).

In December 2023, repeat cystoscopy revealed a new lesion at the bladder neck with persistent gross hematuria and the patient changed his attitude to remove his bladder. The patient underwent digital rectal examination due to elevated prostate-specific antigen (PSA). No suspicious prostate nodules or bilateral inguinal lymph nodes were palpated. As contrast-enhanced abdominal and chest CT showed no metastases, the patient underwent laparoscopic radical cystoprostatectomy with ileal conduit diversion and bilateral pelvic lymphadenectomy. Among 13 dissected pelvic lymph nodes (8 left, 5 right), only one node (1/8 from left side) demonstrated metastatic involvement. This specific lymph node, which measures 14 × 9 mm in maximal cross-section, contains metastases from both urothelial and prostatic carcinoma (**Figure 1A**). Metastasis from urothelial carcinoma accounts for 50% of the lymph node area, while metastasis from prostatic carcinoma accounts for approximately 20%. Final pathology revealed high grade urothelial carcinoma (pT2bN1) characterized by localized glandular differentiation (**Figure 1B, D**) and prostatic adenocarcinoma (pT2N1, Gleason score 4 + 4 = 8) (**Figure 1C**).

Immunohistochemical analysis of the primary tumor in bladder and prostatic showed: Bladder tumor: CK20 (+), 34βE12 (+), p63 (focal+), p53 (+), HER2 (+++), EMA (+), Ki-67 (50%+ in hotspot areas), CK7 (majority+), PSA (−), Vim (−), P40 (−). Prostate tumor: P504S (+), PSA (+), CD34 (vascular+), D2-40 (lymphatic+), S-100 (neural+); 34βE12 (−), p63 (−), Ki-67 (5%+ in hotspot areas). We also performed immunohistochemical staining on the metastatic foci in the metastatic lymph node. The collision metastasis in this lymph node was confirmed by immunohistochemical staining for CK7 (**Figure 1E**), CK20 (**Figure 1F**), GATA3 (**Figure 1G**), PSA (**Figure 1H**), and P504S (**Figure 1I**).

### 2.2 Postoperative adjuvant therapy

Preoperative evaluation demonstrated a prostate volume of 34 ml by CT imaging and serum PSA level was 10.88 ng/ml. in December 2023. Following RC, the PSA declined to 0.04 ng/ml by January 2024. As lymph node metastasis was found in this patient, Androgen deprivation therapy (ADT) was given to this patient. Subsequent PSA dynamics after ADT are presented in **Supplementary Materials**. The patient also received chemotherapy in February 2024 with a regimen of gemcitabine 1.6g(850 mg/m², once at day 1 and day 8), combined with cisplatin 100 mg (52 mg/m² at day 2), administered every 3 weeks. After two cycles of this chemotherapy, due to myelosuppression and gastrointestinal adverse reactions, the patient commenced immunotherapy with Toripalimab 240 mg, a PD-1 antibody approved in China for urothelial cancers, every 21 days in April 2024.

**Figure 2:**
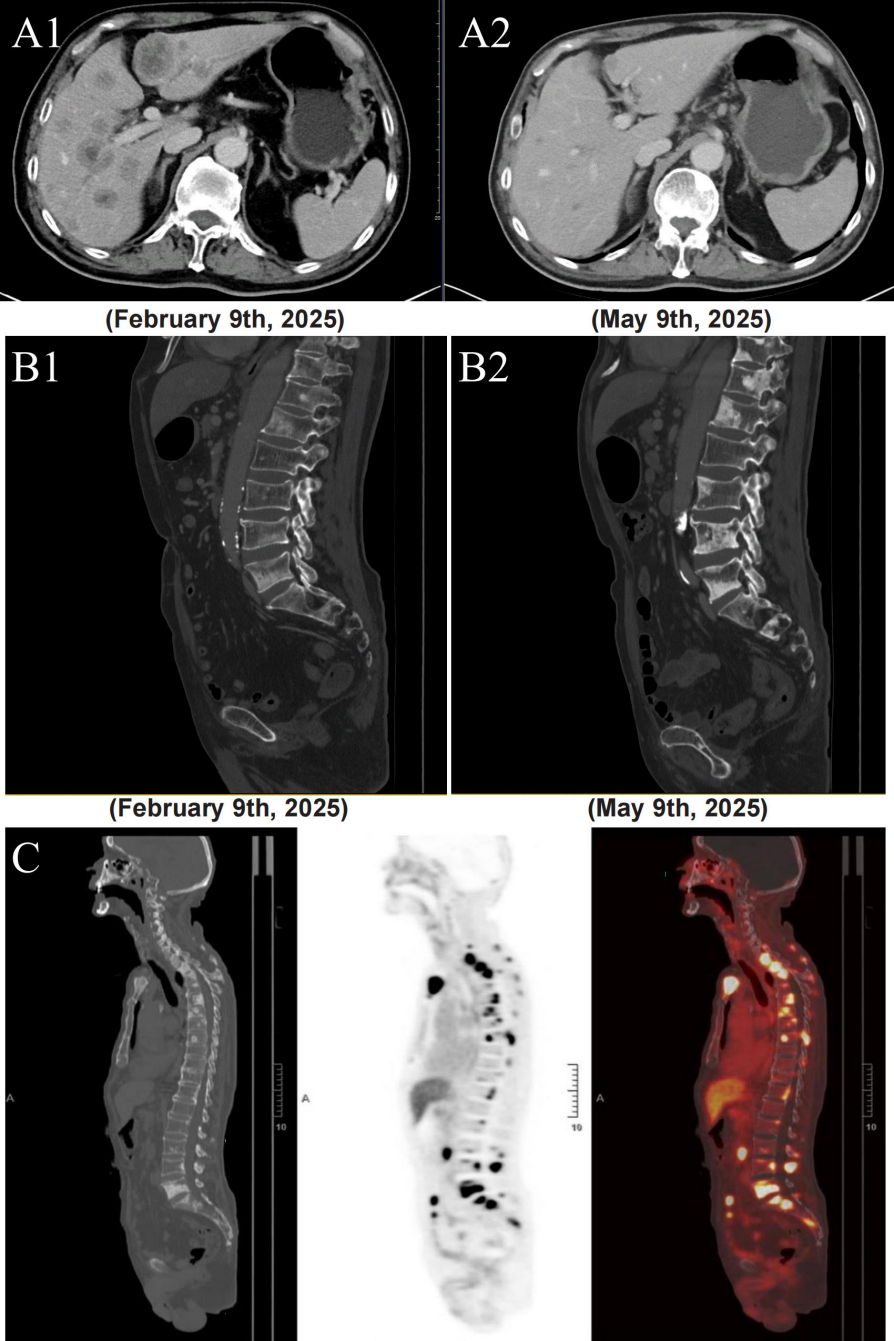
**(A1, B1)** Multiple metastatic tumors in the liver and bone shown in the enhanced abdominal CT scan. Before the treatment with Disitamab Vedotin. **(A2, B2)** After three treatments with 120 milligrams of Disitamab Vedotin. **(C)**^18^F-PSMA PET/CT demonstrating radiotracer uptake in liver (SUV max=7.52) and bone (SUV max=14.33).

In February 2025, the patient presented to the clinic due to worsening right hip pain. A plain chest CT scan and an abdominal contrast-enhanced CT scan were performed, revealing multiple bone metastatic lesions (involving the ribs, thoracic vertebrae, lumbar vertebrae, constituent bones of the pelvis, and the right femoral trochanter) as well as multiple hepatic metastatic lesions. Given the immunohistochemical finding of HER2 (+++) in the bladder tumor, the patient began to receive Disitamab Vedotin 120 mg, an ADC targeted for HER2 and approved in China for urothelial cancers, every 3 weeks. Concurrently, bone-protective treatment was implemented by using Zoledronic acid at a dosage of 4 mg every 3 weeks. Following three cycles of Disitamab Vedotin treatment, an abdominal contrast-enhanced CT scan performed in May 2025 revealed a substantial decrease in hepatic lesions compared with the abdominal contrast-enhanced CT scan conducted in February 2025; however, an increase in bone lesions was observed (**Figures 2A1, 2, B1, 2**). The patient declined bone biopsy and then a ^18^F-prostate-specific membrane antigen (PSMA)-PET/CT examination was performed to this patient. Examination results showed multiple metastatic lesions in the liver and bones. The bone metastatic lesions were predominantly osteoblastic in nature. The maximum standardized uptake value (SUV max) was 7.52 for the liver and 14.33 for the bones (**Figure 2C**).

## 3 Discussions

Historically, different authors have had slightly varying definitions of “collision tumors”, with one point of difference being whether a transitional pattern is allowed to exist in the collision area. In 1961, Dodge (1) proposed that a “collision tumors” refers to two tumors independently originating from different sites, which need to meet the following conditions: the presence of two separate tumor regions with distinct histological characteristics; if both types of tumors metastasize, they should exhibit clearly distinguishable growth patterns in the metastatic lesions, and there should be no transitional areas between them. In 1980, Spagnolo and Heenan (2) proposed that the diagnosis of “collision tumors” should be based on two essential criteria: anatomically, the two components must originate from distinct sites; and there should be at least some degree of histological separation between the closely adjacent components. The observation of transitional patterns at the collision zone is permissible, as they hypothesized that during tumor progression, the two components may invade each other, consequently forming an intermediate zone with hybrid histopathological features. In 2009, Satter et al. (3) defined collision tumors as: “two independent neoplasms that occur in close proximity to one another, yet maintain sharply distinct boundaries.” In their 2020 review on the types, pathogenesis, and diagnostic challenges of collision tumors, Bulte et al. (4) endorsed the definition proposed by Satter et al.

Possible explanations for collision metastases of malignant tumors within the same lymph node include: (i) Certain cancers, such as prostate cancer, tend to have high incidence and lengthy disease progression, often presenting subtle symptoms. This long timeline provides ample opportunity for a second primary tumor to emerge and spread to the same lymph node (5, 6). (ii)Some researchers have proposed the “altered tumor microenvironment” hypothesis: the first metastatic tumor may modify the local microenvironment to facilitate colonization and growth of a second tumor (7), this mechanism may similarly apply to lymph node collision metastases. (iii)Gasparinho et al. (8) documented a case of metastatic collision within a lymph node involving both breast carcinoma and rectal neuroendocrine carcinoma. They subsequently proposed the “chemokine-guided migration” hypothesis, suggesting that malignancies with comparable chemokine receptor expression profiles may exhibit tropism toward specific lymph nodes or organs, potentially resulting in collision metastases.

The incidental detection of prostate cancer in radical cystoprostatectomy specimens represents a relatively common clinical phenomenon. Baio et al. (9) conducted a single-center retrospective study that analyzed data from 303 patients with urothelial carcinoma of the bladder who underwent radical cystoprostatectomy (excluding patients with a preoperative diagnosis or clinical suspicion of prostate cancer). Among these, 69 patients (22.7%) were found to have prostate cancer. Wu et al. (10) conducted a population-based cohort study involving 14,199 bladder cancer patients who underwent radical cystoprostatectomy. Among these patients, 4,096 (28.8%) were incidentally diagnosed with prostate cancer. Notably, 89.9% of these prostate cancer cases were localized (stage T1-2).

However, the phenomenon of collision metastases within the same lymph node from coexisting bladder and prostate cancers is exceptionally rare. To our knowledge, there are only seven documented cases of collision metastases involving urothelial carcinoma and prostatic adenocarcinoma in the literature (**Table 1**). These tumors may display similar morphological features and cannot be clearly differentiated solely through HES staining. Consequently, immunohistochemistry is necessary for distinguishing between poorly differentiated prostatic adenocarcinoma and urothelial carcinoma. Among the seven cases, PSA antibodies were used in six cases, a combination of CK7 and CK20 antibodies in three, and GATA3 antibody in two. In our specific case, staining with PSA, CK7, CK20, and GATA3 antibodies successfully differentiated the two tumor components in the lymph node. However, staining with the P504S antibody showed positive results in both tumor components. Langner et al. (17) demonstrated P504S expression in 127 of 261 urothelial carcinomas (48.7%), with its positivity rate showing positive correlation with tumor stage and grade. In this case, we observed more intense P504S antibody staining in the prostatic adenocarcinoma metastatic foci compared to the bladder carcinoma foci within the same lymph node (**Figure 1I**).

**Table 1:** Reported cases of collision metastases involving urothelial carcinoma and prostatic adenocarcinoma in lymph nodes.

Author	Ergen et al.	Gohji et al.	Overstreet et al.	Bhavsar et al.	Junca et al.	Sellman et al.	Buzoianu et al.	Present case
year	1995 (11)	1997 (12)	2001 (13)	2012 (7)	2015 (14)	2018 (15)	2024 (16)	2025
Country	America	Japan	America	America	France	America	Romania	China
Age	67	78	67	83	61	73	64	76
Histological Type of Urothelial carcinoma	High grade Bladder TCC	Moderately grade Bladder SCC	High grade Bladder TCC	High grade Bladder TCC	High grade Ureter TCC	High grade Bladder TCC	High grade Bladder TCC	High grade Bladder TCC (local glandular differentiation)
Preoperative PSA Level (ng/ml)	10.3	11.2	Not specified	Not specified	12	26	5.2	10.9
Total LN (surgically removed)	6 (obturator LN)	16	Not specified	Not specified	6	40	8	13
Collision LN Metastases	1	5	1	1	1	1	1	1
TN of Urothelial carcinoma	pT2a pN2	Not specified	pT4	pT3 pN2	pT2 pN2	pT3a pN2	pT3b	pT2b pN1
TN of prostatic carcinoma	Not specified	Not specified	pT2 pN1	pT3a pN1	pT3b pN1	pT3b pN1	pT3b pN1	pT2 pN1
PCa Gleason Score	4	Not specified	7(3 + 4)	8(4 + 4)	7(3 + 4)	8(4 + 4)	7(4 + 3)	8(4 + 4)
Antibodies Used in IHC	Not specified	PSA	CK20, CK7, PSA, PAP, mACE, CD57	Pan-CK, CK7, CK20, PSA	CK7, CK20, PSA, RA GATA3, PIN-Cocktail,	CK7, CK20, p40, PSA	CK19, p40, GATA3, PSA	CK7, CK20, PSA, GATA-3, P504S

TN, the 2017 UICC TNM; LN, lymph node; IHC, immunohistochemistry; SCC, squamous cell carcinoma; TCC, transitional cell carcinoma.

Among seven reported cases in literature, most cases did not mention postoperative treatment and follow-up data. In our case, the liver metastases responded effectively to Disitamab Vedotin treatment, supporting the notion that the tumor originated from urothelial carcinoma. However, the bone metastases progressed after treatment, suggesting that they might not be derived from urothelial carcinoma. The efficacy of Disitamab Vedotin in treating HER2-overexpressing urothelial carcinoma has been clinically validated. Sheng et al. (18) performed a pooled analysis of two Phase II clinical trials, involving 107 patients with HER2-positive locally advanced or metastatic urothelial carcinoma whose disease had progressed despite at least one prior systemic chemotherapy regimen. These patients received Disitamab Vedotin treatment at a dose of 2 mg/kg every two weeks. The overall confirmed objective response rate was 50.5% (95% CI, 40.6% to 60.3%). Among these patients, there were 48 cases each with liver metastases and bone metastases. However, the analysis did not address the “dissociated responses” of these two types of metastases during the course of treatment.

Anti-HER2 therapy demonstrates limited efficacy and lacks robust evidence in metastatic prostate cancer, primarily due to inconsistent outcomes and inadequate clinical responses (19, 20). The presence of osteoblastic lesions and a SUV max of 14.33 on ^18^F-PSMA-PET/CT suggests that the bone metastases may originate from prostate cancer.

In the present case, the patient was diagnosed with two distinct malignant neoplasms requiring systemic therapy: urothelial carcinoma and prostate cancer. The disease progression was characterized by initial nodal collision metastases, followed by the subsequent development of multiple metastatic lesions in the liver and bones. However, these two malignancies have distinct first-line standard treatment regimens. Specifically, for urothelial carcinoma, the recommended first-line options include Enfortumab Vedotin plus Pembrolizumab or platinum-based chemotherapy (e.g., cisplatin plus gemcitabine). In contrast, for metastatic prostate cancer, the standard first-line therapeutic strategies consist of antibody-drug conjugate (ADC) combined with novel hormonal therapy, as well as triple therapy regimens comprising ADT plus docetaxel and darolutamide/abiraterone. The ideal therapeutic strategy for such patients is to identify a drug combination that exerts efficacy against both tumor components while exhibiting non-overlapping or minimally overlapping toxicity profiles. This approach aims to maximize therapeutic benefits while controlling toxicities within a manageable range. Chen et al. (21) reported a case of collision carcinoma involving lung adenocarcinoma and prostate cancer. Specifically, the postoperative pathological stage of lung adenocarcinoma was pT1N2M0 with an EGFR exon 19 deletion mutation, while the prostate cancer was complicated by multiple metastases to the lungs, lymph nodes, and bones. Both adjuvant chemotherapy and targeted therapy are recommended as first-line treatment options for the patient’s lung cancer. The patient initially initiated treatment for prostate cancer with a regimen consisting of goserelin acetate combined with abiraterone acetate. Concurrently, ibandronate sodium was administered for anti-bone metastasis therapy. Targeted therapy with osimertinib (80 mg, once daily) was initiated three weeks after lung cancer surgery. Following the operation, the patient underwent regular re-evaluations and maintained treatment with the aforementioned regimen. The tumors were well-controlled, with no evidence of recurrence observed. Sellman et al. (15) identified a case of collision metastasis involving bladder urothelial carcinoma and prostatic adenocarcinoma in a pelvic lymph node following radical cystoprostatectomy. They elected to promptly initiate adjuvant hormonal therapy combined with a chemotherapy regimen consisting of Cisplatin and Gemcitabine. During the initial follow-up period, no evidence of disease progression was observed. In the present case, a platinum-containing regimen was administered for the high-grade urothelial carcinoma, while ADT was initiated for the management of prostate cancer. Upon disease progression, a HER2-targeted antibody-drug conjugate (ADC) combined with a PD-1 inhibitor was administered to control liver metastases derived from urothelial carcinoma. Concurrently, novel hormonal therapy agents and denosumab were added to treat bone metastases originating from prostate cancer. Considering the patient’s tolerability, taxane-based chemotherapy was not utilized. Clinical follow-up data demonstrated that the patient currently maintained favorable disease control with a high quality of life.

To further understanding “collision metastasis” in lymph nodes, we conducted comprehensive searches across multiple databases including PubMed, China National Knowledge Infrastructure, Chinese Medical Journal Full-text Database, and Wanfang Database. We categorized and analyzed 32 reported cases of lymph node collision metastasis. Literature reports describing the coexistence of metastatic tumors and lymphoproliferative disorders within the same lymph node were excluded, as these cases ought to be classified as inter-tumor metastasis (**Table 2**). The most frequently involved sites were pelvic lymph nodes (9/32 cases, 28.1%) and cervical lymph nodes (10/32 cases, 31.3%). Regarding primary malignancies, prostate cancer accounted for 17 cases (53.1%), while thyroid cancer comprised 10 cases (31.3%), including 3 cases (9.4%) demonstrating collision metastases between two distinct histological subtypes of thyroid carcinoma.

**Table 2:** Reported cases of collision metastases in the same lymph node.

Nodal Site	Primary Tumors	Age	Sex	Author	Year
Pelvic	Prostatic adenocarcinoma/Urothelial bladder tumor	67	M	Ergen et al. (11)	1995
	Prostatic adenocarcinoma/Urothelial bladder tumor	67	M	Overstreet et al. (13)	2001
	Prostatic adenocarcinoma/Urothelial bladder tumor	83	M	Bhavsar et al. (7)	2012
	Prostatic adenocarcinoma/Urothelial bladder tumor	61	M	Junca et al. (14)	2015
	Prostatic adenocarcinoma/Urothelial bladder tumor	73	M	Sellman et al. (15)	2018
	Prostatic adenocarcinoma/Urothelial bladder tumor	64	M	Buzoianu et al. (16)	2024
	Prostatic adenocarcinoma/Urothelial bladder tumor	76	M	Present case	2025
	Prostatic adenocarcinoma/Epidermoid bladder tumor	78	M	Gohji et al. (12)	1997
	Melanoma/Urothelial bladder tumor	82	M	Sanguedolce et al. (22)	2022
Cervical	Papillary thyroid carcinoma/Medullary thyroid carcinoma	41	M	Pastolero et al. (23)	1996
	Papillary thyroid carcinoma/Medullary thyroid carcinoma	32	M	Sadat Alavi et al. (24)	2012
	Papillary thyroid carcinoma/Medullary thyroid carcinoma	32	M	Zhao et al. (25)	2025
	Papillary thyroid carcinoma/Squamous tongue carcinoma	51	M	Guelfucci et al. (26)	2004
	Squamous oral carcinoma/Thyroid carcinoma	57	M	Elias da Cruz Perez et al. (27)	2008
	Papillary thyroid carcinoma/Squamous oral carcinoma	47	M	Lim et al. (28)	2008
	Papillary thyroid carcinoma/Squamous oral carcinoma	63	M	Xu et al. (29)	2018
	Papillary thyroid carcinoma/Squamous thyroid carcinoma	73	F	Alhanafy et al. (30)	2016
	Papillary thyroid carcinoma/Squamous cell carcinoma (unknown primary tumor)	50	F	Mattioli et al. (31)	2009
	Papillary thyroid carcinoma/Ductal breast carcinoma	49	F	Zeng et al. (32)	2012
Perirectal	Prostatic adenocarcinoma/Rectal adenocarcinoma	72	M	Morgan et al. (33)	1969
	Prostatic adenocarcinoma/Rectal adenocarcinoma	61	M	Wade et al. (6)	2004
	Prostatic adenocarcinoma/Rectal adenocarcinoma	70	M	Mourra et al. (34)	2005
	Prostatic adenocarcinoma/Rectal adenocarcinoma	82	M	Miyauchi et al. (35)	2013
Thoracic	Esophageal adenocarcinoma/Ductal breast carcinoma	51	F	El-Gendy et al. (36)	2008
	Neuroendocrine rectal carcinoma/Ductal breast carcinoma	55	F	Gasparinho et al. (8)	2011
	lung adenocarcinoma/Prostatic adenocarcinoma	71	M	Chen et al. (21)	2022
Axillary	Serous papillary ovarian carcinoma/Ductal breast carcinoma	62	F	Sughayer et al. (5)	2009
	Prostatic adenocarcinoma/Melanoma	71	M	Saco et al. (37)	2018
Mesenteric	Prostatic adenocarcinoma/Colonic adenocarcinoma	80	M	Wade et al. (6)	2004
Para-aortic	Prostatic adenocarcinoma/Gastric adenocarcinoma	83	M	Terada et al. (38)	1993
Retroperitoneum	Prostatic adenocarcinoma/Renal cell carcinoma	50	M	Morton et al. (39)	2022
Peri-parotid	Melanoma/Squamous cutaneous carcinoma	79	M	Wu et al. (40)	2024

The present study has the following limitations: the follow-up duration of the case is relatively short, resulting in a lack of survival data; in addition, the hepatic and osseous metastatic lesions of this patient lack pathological evidence and are solely based on limited inferences derived from imaging findings and treatment responses, which inherently entails uncertainty.

## 4 Conclusion

When markedly distinct histological morphologies are observed in lymph node metastases, the possibility of collision metastases must be considered to avoid missing a second tumor type. Immunohistochemical staining serves as a crucial tool for differentiating various collision tumors. Lymph node collision metastases represent a special type of lymph node metastasis. Research into their molecular mechanisms may yield novel insights into tumor lymph node metastasis and advance our understanding of the competitive metastatic mechanisms among different cancer types within the tumor microenvironment.

## Data Availability

The original contributions presented in the study are included in the article/**Supplementary Material**. Further inquiries can be directed to the corresponding authors.

## Supplementary material

Supplementary Figure 1 The dynamic changes in serum PSA levels of this patient from January 2023 to June 2025.

Supplementary Figure 2 **(A)** Sep. 2023 TURBT Specimen (Urothelial Carcinoma)- HER2 IHC. **(B)** Dec. 2023 RC Specimen (Urothelial Carcinoma) - HER2 IHC.
