# Supplementary figures and images for: Collision metastasis in a pelvic lymph node from urothelial and prostatic carcinoma: a rare case report and literature review
Source: Front Oncol. 2025 Dec 4;15:1658290. doi: 10.3389/fonc.2025.1658290 (PMC12711506; doi:10.3389/fonc.2025.1658290)

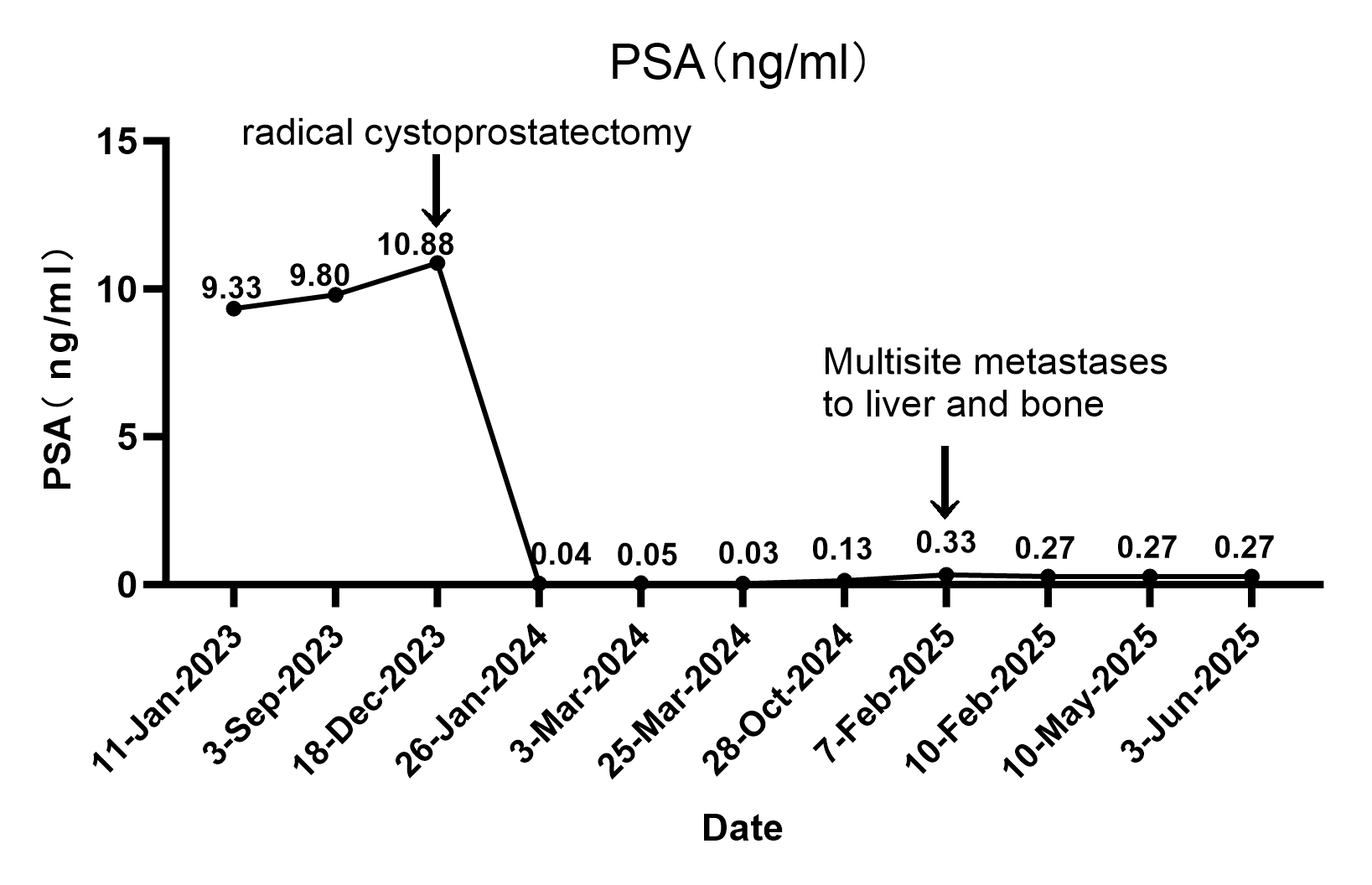

Supplement: Supplementary Figure 1 — The dynamic changes in serum PSA levels of this patient from January 2023 to June 2025. [file Image1.tif]

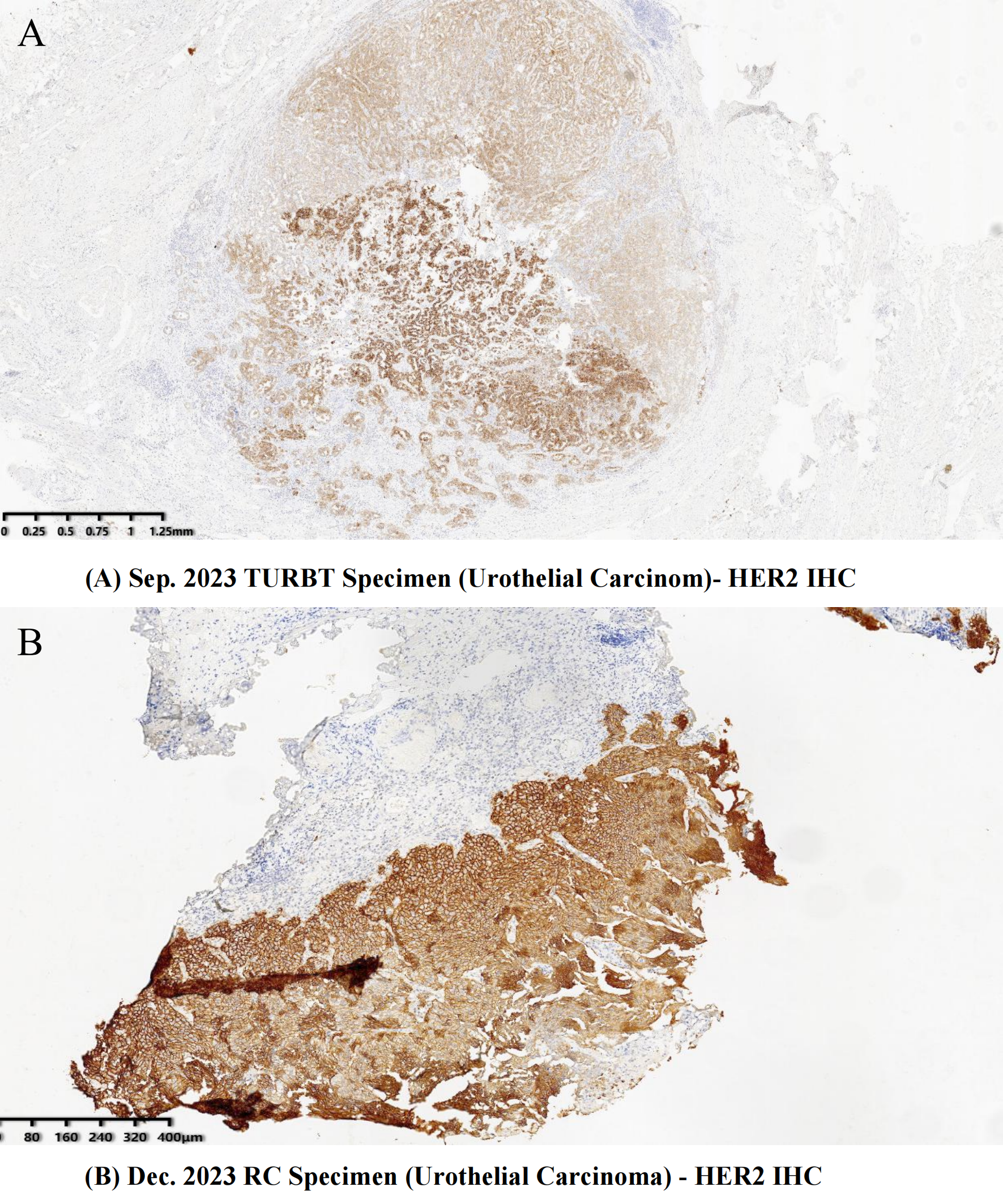

Supplement: Supplementary Figure 2 — (A) Sep. 2023 TURBT Specimen (Urothelial Carcinoma)- HER2 IHC. (B) Dec. 2023 RC Specimen (Urothelial Carcinoma) - HER2 IHC. [file Image2.tif]
